# Outcomes of Liver Transplantation in Patients With Congenital Heart Disease and Biliary Atresia. A Multicenter Analysis

**DOI:** 10.1111/petr.70110

**Published:** 2025-05-30

**Authors:** Mario O'Connor, Joel T. Adler, Neil M. Venardos, Monica S. Ponce‐Rivera, Charles D. Fraser, Carlos M. Mery, Andrew Well

**Affiliations:** ^1^ Department of Surgery and Perioperative Care Dell Medical School at the University of Texas, Austin Austin TX USA; ^2^ Department of Pediatrics Dell Medical School at the University of Texas, Austin Austin TX USA; ^3^ Texas Center for Pediatric and Congenital Heart Disease Dell Children's and UT Health, Texas, Austin Austin TX USA; ^4^ Department of Surgery and Perioperative Care, Division of Transplant Surgery Dell Medical School at the University of Texas at Austin Austin TX USA

**Keywords:** biliary, congenital liver disease, extrahepatic bile duct atresia, liver transplant, outcome

## Abstract

**Introduction:**

Congenital heart disease (CHD) frequently coexists with noncardiac malformations. Among which, biliary atresia (BA) occurs in approximately 5%–15% of patients Despite this, outcomes of liver transplantation (LT) in patients with CHD and BA remain unknown.

**Methods:**

A retrospective review of the Pediatric Health Information System (PHIS) database from January 1, 2004 to October 30, 2023. All patients who underwent LT and had a diagnosis of BA were included. Multiorgan transplants were excluded. International Classification of Diseases 9th and 10th editions were utilized to identify patients with a diagnosis consistent with CHD.

**Results:**

A total of 1677 patients were identified with 983 (59%) female, 811 (5%) white non‐Hispanic, and a median age at transplant of 11.6 (interquartile range [IQR]: 7.7–26.6) months. A CHD diagnosis was present in 83 (5%). Overall, the majority of transplants were performed in the CHD population in the modern era (2016–2023) (42/83.51%). CHD had a longer median preoperative length of stay (LOS) compared to non‐CHD (1 [1.0–15.0] vs. 1 [0–6.0], *p* = 0.031). No increased risk of in‐hospital mortality was evident (OR: 1.61, 95% CI: 0.37–6.95, *p* = 0.519). On multivariable analysis, CHD was associated with a 29% (95% CI: 11.04–50.34) increase in LOS (*p* < 0.001), 25% (95% CI: 6.98–46.47, *p* = 0.005) increase in postoperative LOS, and was not associated with increased risk for 30‐day readmission (OR: 1.14; CI: 0.53–2.45, *p* = 0.736). Over a median follow‐up of 3.5 years (IQR: 0.86–7.78) years, no difference in retransplantation rate was evident.

**Conclusions:**

LT in patients with CHD and BA is safe. Although patients with CHD display heightened utilization of in‐hospital resources, no discernible variance in long‐term outcomes was observed. However, additional research is imperative to comprehensively elucidate the influence of CHD on management decisions and outcomes throughout the liver transplant process.

## Graphical abstract

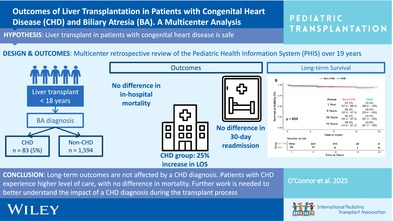

## 1 Introduction

Liver transplantation (LT) is the gold standard treatment for patients with end‐stage liver disease [1]. Biliary atresia (BA) and metabolic disorders represent the most common reasons for LT in the pediatric population [1, 2]. In patients with BA, 15%–30% can present with extrahepatic congenital abnormalities [3, 4, 5].

A congenital heart disease (CHD) diagnosis is present in 5%–15% of patients with BA [3, 6, 7]. The most common CHD diagnoses include atrial septal defect (ASD), ventricular septal defect (VSD), patent ductus arteriosus (PDA), and pulmonary stenosis or atresia [6, 7, 8]. With improvement in survival of patients with CHD, more patients are undergoing non‐CHD‐related procedures; thus, understanding the implication of CHD on LT outcomes becomes relevant [9].

Historically, cardiopulmonary anomalies and pulmonary hypertension were viewed as contraindications for LT [10]. However, advancements in care and timely interventions for patients with CHD have reduced these complications [11, 12]. Despite these improvements, intrinsic cardiovascular changes, along with intraoperative and postoperative hemodynamic fluctuations, can still significantly impact outcomes in this population.

Liver disease inherently affects cardiovascular function, leading to reduced systemic vascular resistance and impaired cardiac contractility [13]. At the time of surgery, LT places significant stress on the cardiovascular system with the associated shift in volumes, decreased venous return, and cardiac output [14, 15]. Thus, CHD represents a challenge not only preoperatively but also during the operative period and postoperatively, as a higher frequency of decompensation has been reported in this population [6].

Current data on the impact of CHD in LT outcomes is limited to case reports and single‐center studies across a variety of liver diseases [6, 8, 16]. Due to the higher prevalence of CHD in patients with BA, this study aims to evaluate the impact of CHD on short‐term and long‐term outcomes of LT for BA using a multi‐institutional database.

## 2 Materials and Methods

### 2.1 Data Source

This is a multicenter retrospective review using the Pediatric Health Information System (PHIS) database, from January 1, 2004, to October 30, 2023. PHIS, maintained by the Children's Hospital Association (CHA), encompasses administrative and billing data from 49 pediatric hospitals in the United States, covering around 20% of pediatric hospitalizations nationwide.

Upon submission, data are deidentified and subjected to thorough quality assessments. Each patient is assigned a unique identifier for each hospital visit, allowing for tracking over time within a single facility, but not across different CHA hospitals. PHIS records contain the primary diagnosis, up to 41 additional diagnoses, principal procedures, and up to 41 supplementary procedures. Diagnoses and procedures were coded according to the standard ICD, Ninth Revision (ICD‐9) until the third quarter of 2015. From the fourth quarter of 2015 onward, records have been coded using the Tenth Revision (ICD‐10).

### 2.2 Study Population

All patients in the PHIS database with ICD codes consistent with BA (ICD 9: 751.61 ICD 10: Q44.2) and liver transplant (ICD 9: 50.59 ICD 10: 0FY00Z0) were included in the main cohort. Previously validated ICD codes were utilized to identify patients with a CHD diagnosis or diagnoses [17]. Those patients with a diagnosis consisting of isolated ASD were considered patients without CHD, as this code has been shown to have poor accuracy in administrative datasets [18]. Patients with isolated PDA were also considered patients without CHD, as PDA is frequently reported as a diagnosis but often represents a normal variation due to delayed closure rather than a congenital heart defect. The following were excluded from analysis: events with missing information on the type of admission, sex, age, race, ethnicity, length of stay (LOS), discharge status, admitting diagnosis, and/or principal diagnosis.

### 2.3 Study Outcomes

The primary aim of the study was to assess the incidence and outcomes of LT in patients with BA and CHD. Demographics collected included age, sex, race, ethnicity, and insurance type. Insurance was grouped into Government (Medicare, Medicaid, Tricare, etc.), Private, and Other (Charity, other payer, unknown). Hospitalization outcomes included the need for prolonged mechanical ventilation (defined as longer than 96 h), LOS and in‐hospital mortality, all provided by PHIS.

Patients were tracked over time to evaluate 30‐day readmission rate. Readmissions were categorized as transplant‐related, infection related, and other causes. Finally, patient survival (death after LT) and graft survival (retransplantation) were collected.

### 2.4 Statistical Analysis

Descriptive statistics were reported for demographics, clinical characteristics, and outcomes. Categorical variables are reported as *n*(%). LOS is reported in median [interquartile range (IQR)] days. Chi‐square and Fisher's exact test were utilized to analyze noncontinuous variables, as indicated. Kruskal–Wallis test was utilized to analyze LOS comparison between groups. Multivariable analyses including linear and logistic multivariable regression were utilized to assess associations with CHD and outcomes. Cox regression analysis and Kaplan–Meier curves were utilized. All statistical tests were two‐tailed, and a *p*‐value < 0.05 was considered significant. Statistical analyses were performed using R and RStudio [19].

## 3 Results

### 3.1 Study Population and Demographics

From January 1, 2004, through December 30, 2023, there were 1667 patients who underwent liver transplant with a diagnosis of BA. Of this cohort, 983 (59%) were female, 811 (48%) White non‐Hispanic, 604 (36%) had private insurance, and were a median age of 11.6 (IQR: 7.7–26.6) months at transplant. A total of 83 (5%) patients were identified as having a diagnosis consistent with CHD Table 1. Ninety‐nine CHD diagnoses were identified in these 83 patients, and the three most common CHD lesions were: VSD (*n* = 24, 24%), ASD + VSD (*n* = 14, 14%), and pulmonary artery stenosis (*n* = 12, 12%) Table S1. There were fewer female patients in the CHD group compared to the non‐CHD group (*n* = 39 [47%] vs. *n* = 944 [59%], *p* = 0.036). No difference in median age at transplant between the CHD group and non‐CHD group was evident (13.1 [IQR: 7.1–24.6] months vs. 11.6 [IQR: 7.7–26.6] months, *p* = 0.714]). Race and insurance distributions were not different between groups Table 1.

**TABLE 1:** Demographics.

Variable	Overall (*n* = 1677)	CHD (*n* = 83) (5%)	Non‐CHD (*n* = 1594) (95%)	*p*
Age at Transplant (Months), Median [IQR]	11.6 [7.7–26.6]	13.1 [7.1–24.6]	11.6 [7.7–26.6]	0.714
Sex				
Female	983 (59)	39 (47)	944 (59)	**0.036**
Race				
White non‐Hispanic	811 (48)	47 (57)	764 (48)	0.442
Hispanic	379 (23)	17 (20)	362 (23)	
Black	233 (14)	10 (12)	223 (14)	
Other	254 (15)	9 (11)	245 (15)	
Insurance				
Private	604 (36)	30 (36)	574 (36)	0.524
Government	837 (50)	38 (46)	799 (50)	
Other	236 (14)	15 (18)	221 (14)	
Region				
Midwest	463 (28)	27 (33)	436 (27)	0.543
Northeast	364 (22)	20 (24)	344 (22)	
South	274 (16)	13 (16)	261 (16)	
West	576 (34)	23 (28)	553 (35)	
Era				
2004–2009	244 (15)	11 (13)	233 (15)	0.942
2010–2015	599 (36)	30 (36)	569 (36)	
2016–2023	834 (50)	42 (51)	792 (50)	

*Note:* Bold values represent statistical significance.

### 3.2 Outcomes

#### 3.2.1 Length of Stay

Overall, total median LOS was 20 [IQR: 13.0–36.0] days, with the CHD group having longer LOS when compared to the non‐CHD group (24 [IQR: 17.0–51.0] days vs. 20 [IQR: 13.0–35.0], *p* = 0.003) (Table 2). Patients with CHD also had longer preoperative LOS when compared to patients without CHD (1 [IQR: 1.0–15.0] days vs. 1 [IQR: 0.0–6.0] days], *p* = 0.031). Median postoperative LOS was 17 [IQR: 11.0–26.0] days, with the CHD group having longer post‐operative LOS compared to the non‐CHD group (20 [IQR: 14.0–35.0] days vs. 16 [IQR: 11.0–26.0] days, *p* = 0.003) Table 2. In multivariable analysis, CHD was associated with a 29.2% (95% CI: 11.0%–50.3%) increase in total LOS (*p* < 0.001) Table S2.

**TABLE 2:** In‐Hospital Outcomes.

Variables	Overall (*n* = 1677)	CHD (*n* = 83) (5%)	Non‐CHD (*n* = 1594) (95%)	*p*
Length of stay (Days), Median [IQR]	20 [13–36]	24 [16.5–51]	20 [13–35]	**0.003**
Pre‐operative LOS (Days), Median [IQR]	1 [0–6]	1 [1–15]	1 [0–6]	**0.031**
Postoperative LOS (Days), Median [IQR]	17 [11–26]	20 [14–35]	16 [11–26]	**0.003**
Prolonged Ventilation	631 (38)	38 (46)	593 (37)	0.145
Red Blood Cell Transfusion	678 (40)	38 (46)	640 (40)	0.365
Mortality	26 (2)	2 (2)	24 (2)	0.371

*Note:* Bold values represent statistical significance.

#### 3.2.2 Prolonged Mechanical Ventilation

**FIGURE 1:**
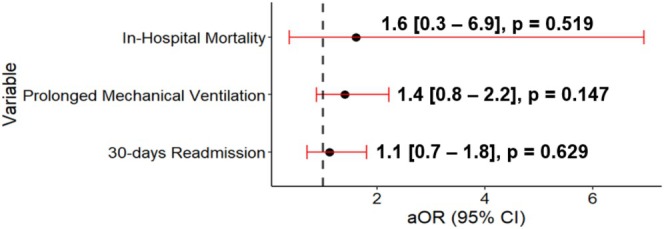
Multivariable logistic regression model results for CHD outcomes compared to non‐CHD. Adjusted for age, race, gender, insurance, era, center volume. aOR, adjusted odds ratio.

A total of 631 (38%) patients had prolonged mechanical ventilation. Of those, 38 (46%) patients with CHD had prolonged mechanical ventilation compared to 593 (37%) in the non‐CHD group. This difference was not statistically significant (*p* = 0.145) (Table 2). In multivariable analysis, when compared to patients without CHD, a CHD diagnosis did not confer increased odds for prolonged mechanical ventilation (OR: 1.40; 95% CI: 0.80–2.20, *p* = 0.147) (Figure 1).

#### 3.2.3 In‐Hospital Mortality

There were 26 (2%) in‐hospital deaths; of those, 2 (2%) occurred in the CHD group compared to 24 (2%) in the non‐CHD group (Table 2). In univariate analysis, a CHD diagnosis did not confer a significant increase in the odds for in‐hospital mortality (OR: 1.61 CI: 0.37–6.95, *p* = 0.519) (Figure 1).

#### 3.2.4 30—Day Readmission

There was a total of 528 (31%) patients who required at least one readmission within 30 days of discharge, 28 (34%) patients in the CHD group and 599 (31%) in the non‐CHD group (*p* = 0.981). There was a total of 738 readmission encounters within 30 days of discharge. Overall, the median number of readmissions per patient within 30 days was 0.0 (IQR: 0.0–1.0). For the entire cohort, the most common reason for readmission within 30 days was transplant related (*n* = 290, 39%). In the multivariable logistic regression model, a CHD diagnosis continued to have no significant association with 30‐day readmission (OR: 1.12; 95% CI: 0.70–1.80, *p* = 0.629) (Figure 1).

### 3.3 Long‐Term Outcomes

#### 3.3.1 Survival

**FIGURE 2:**
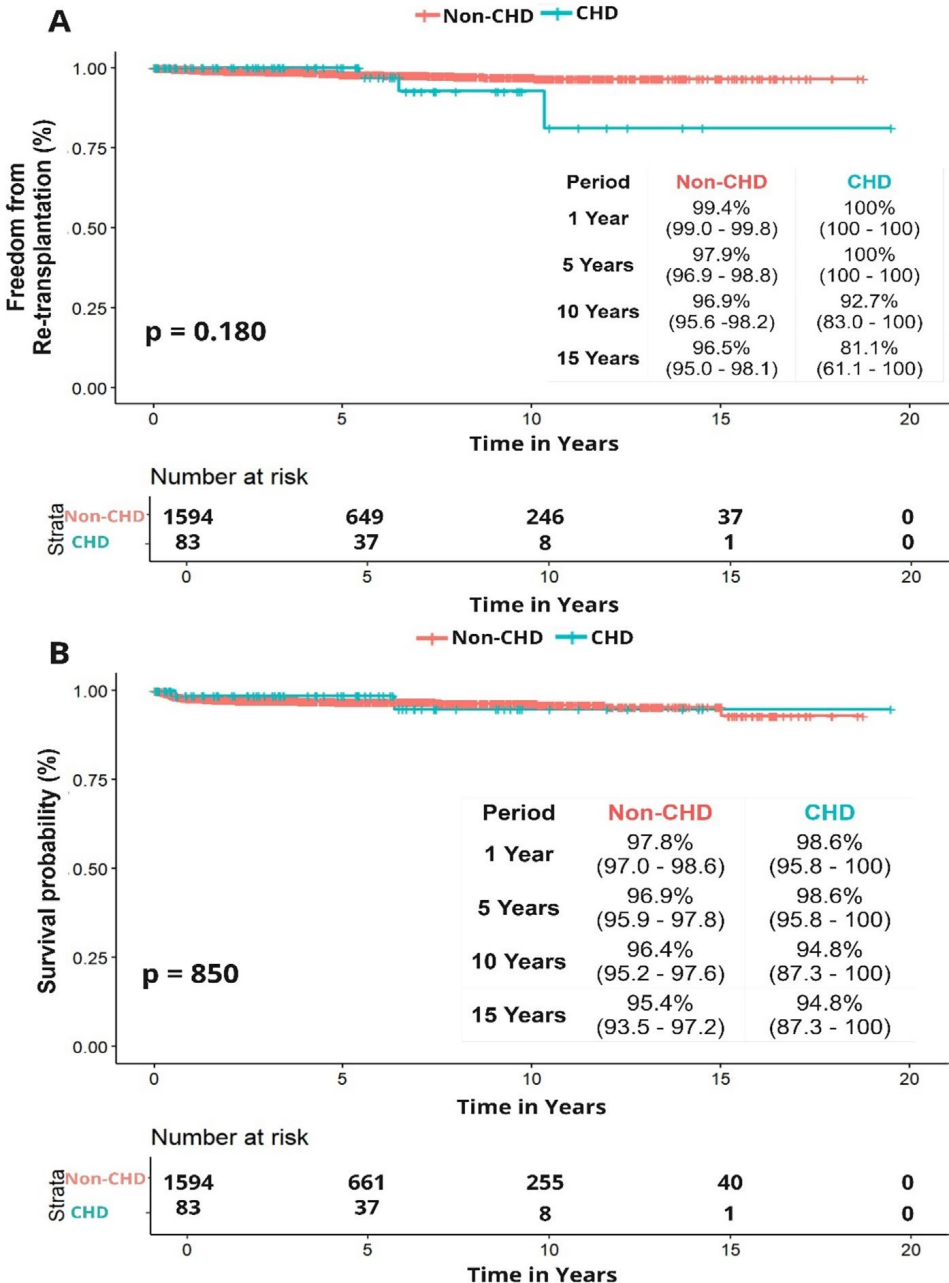
Freedom from (A) re‐transplantation and (B) survival. Liver transplantation in patients with CHD and BA is safe. Although patients with CHD display heightened utilization of in‐hospital resources, no discernible variance in long‐term outcomes was observed.

Over a median follow‐up of 3.5 (IQR: 0.8–7.7) years, there were a total of 46 deaths. The median time from transplant to death was 16 (IQR: 10.6–45.2) months. Two (2%) patients died in the CHD group and 44 (3%) in the non‐CHD group. When comparing patient survival using the Kaplan–Meier method, patient survival was not statistically significantly different between groups (log‐rank *p* = 0.850). The 1‐ and 15‐year patient survival in the CHD group versus non‐CHD group was 98.6% versus 97.8% and 94.8% versus 95.4%, respectively (Figure 2).

In univariate Cox regression analysis, a CHD diagnosis was not associated with increased mortality (HR: 0.87; 95% CI: 0.21–3.61, *p* = 0.855). After adjustment of potential confounders, a CHD diagnosis continued to not be significantly associated with mortality (HR: 0.80; 95% CI: 0.19–3.31, *p* = 0.758).

#### 3.3.2 Graft Survival

During the study period, 29 (2%) patients underwent liver re‐transplantation, 3 (4%) in the CHD group and 26 (2%) in the non‐CHD group. Median time to re‐transplantation was similar between both groups and not statistically significant (4.2 [IQR: 1.10–6.70] years vs. 3.40 [IQR: 0.80–7.70] years, *p* = 0.722). When comparing graft survival using the Kaplan–Meier method, graft survival was not statistically significantly different between groups (log‐rank *p* = 0.180). The 1‐and 15‐year graft survival in the CHD group versus non‐CHD group was 100% versus 99.4% and 81.1% versus 96.5%, respectively (Figure 2).

In univariate Cox regression analysis, a CHD diagnosis did not increase risk for re‐transplantation when compared to the non‐CHD (HR: 2.21; 95% CI: 0.66–7.32, *p* = 0.193). After adjustment for confounders, a CHD diagnosis remained nonsignificant for risk for retransplantation (HR: 2.00; 95% CI: 0.60–6.64, *p* = 0.253).

## 4 Discussion

This study utilizes data from PHIS over 19 years to investigate the incidence, short‐term outcomes, and long‐term outcomes of LT in patients with BA and CHD. Among the study cohort, 83 (5%) patients with BA and a CHD diagnosis underwent LT. Overall, our study found that patients with CHD have increased in‐hospital resource utilization compared to patients without CHD, with a prolonged LOS, but no impact on mortality and long‐term outcomes.

Previous studies on outcomes of LT in patients with CHD are limited to case reports or single‐center studies, and across a variety of liver diseases [6, 8, 16]. Thus, to the best of our knowledge, this study represents the largest study to date on LT outcomes in patients with BA and CHD.

Patients with BA are at increased risk for CHD. Several studies have reported that between 5% and 15% of patients with BA have a diagnosis of CHD [3, 7, 20]. In this study, we report a prevalence of 5% of CHD in patients with BA undergoing LT. This study excludes patients with isolated ASD and isolated PDA; thus, the prevalence of CHD in patients with BA undergoing LT may be higher. This is particularly true for patients with genetic syndromes, where CHD may be more common [21]. However, by excluding these isolated lesions, we present a cohort of patients with CHD with more complex lesions compared to previous reports, where 30% or more of their CHD cohorts are constituted by patients with isolated ASD and isolated PDA [6, 8, 22]. While we present a more complex cohort of patients with CHD, we are potentially excluding other patients with more complex CHD diagnoses that underwent combined heart‐liver transplant and those that were not considered candidates for LT. As such, further work is needed to understand the impact of CHD in the LT process and willingness to transplant.

LT is marked by significant hemodynamic instability [15]. Anesthesia induction, fluid shifts, and blood loss during dissection contribute to this instability. Furthermore, retraction of the inferior vena cava and clamping can reduce venous return and cardiac output [15]. Additionally, postreperfusion syndrome can contribute to the greatest impairment by compromising myocardial contractility, heart rate, and peripheral vascular resistance [23]. Given the mentioned factors, managing the perioperative and postoperative stress is challenging, especially for patients with CHD, as some lesions may present with compromised hemodynamics, making it harder to cope with these additional stressors.

In our study, patients with CHD were more likely to have prolonged post‐transplant LOS; this is in line with previous reports where patients with more simple CHD diagnoses had longer intensive care unit and total LOS [22]. These results could be either a reflection of a more challenging postoperative period due to potential hemodynamic instability, worse clinical status at transplant, or institutional preferences for monitoring patients with CHD for a longer period. Research on factors associated with prolonged LOS after LT in patients with CHD is needed to identify possible interventions, as longer LOS has been associated with worse long‐term outcomes [24]. Additionally, patients with CHD experienced a prolonged pretransplant LOS, which may be attributed to a longer wait for a suitable donor, the need for cardiac interventions, and clinical instability that could render them ineligible for surgical procedure at a certain point in time. With the current data, we cannot explain this; therefore, further work should focus on identifying factors associated with a longer pretransplant LOS in patients with CHD.

Despite more intensive care and potentially higher surgical risk for patients with CHD, outcomes for the entire cohort were good. Notably, there were only 26 (2%) deaths in the entire cohort, 2 (2%) in the CHD group, and 24 (2%) in the non‐CHD group. Our study shows better results with lower mortality, compared to current data where mortality in patients with simpler CHD lesions undergoing LT for several indications is reported between 7% and 15% [6, 8]. While no difference in mortality between groups was evident, the administrative data limit our ability to understand the cause of death of those patients; thus, more studies are needed to better understand the impact and causes of in‐hospital mortality in patients with CHD.

There is no national baseline readmission rate available for pediatric transplant programs, leaving us without a means of general comparison. However, single center experiences have reported 30‐day readmission rates between 26% and 58% [25, 26, 27]. In our analysis, 31% of our cohort had at least one readmission during the first 30 days postdischarge, which is in line with these previous reports. No difference in readmissions or reason for readmission between the CHD group and non‐CHD group was evident. Further studies are needed to evaluate the level of care associated with these readmissions and to determine if patients with CHD require a higher level of care and its financial burden.

Over the past years, significant advances have been made in the pre‐, intra‐, and postoperative management of LT, improving long‐term outcomes [28, 29]. Survival in patients with BA depends primarily on the outcomes of LT [30]. Overall, graft survival in the non‐CHD population is reported to be between 75% and 91% at 20 years post‐transplantation, while overall patient survival after LT is between 85% and 95% at 15 years post‐transplantation [31, 32, 33, 34]. In our analysis, over a median follow‐up of 3.5 years (IQR: 0.86–7.78) no difference in retransplantation rates between CHD and non‐CHD was found. Freedom from retransplantation at 15 years was 81.1% in the CHD group and 96.5% in the non‐CHD group. Patient survival in our cohort at 15 years was 94.8% in the CHD group and 95.4% in the non‐CHD group, which is in line with these previous reports and suggests that LT in patients with BA is effective and safe.

While our study did not find any differences in long‐term outcomes between patients with CHD and patients without CHD, Li et al. reported that graft survival was lower and mortality higher in patients with CHD when compared to patients without CHD [8]. In contrast, another single‐center study reported comparable outcomes between both groups [22]. This suggests potential variability in outcomes across centers and highlights the need for further investigation into the patient selection and management strategies for patients with CHD undergoing LT to reduce these differences. While no differences were found in these single‐center studies, patients with CHD were found to have an increased risk for cardiac decompensation both before and after LT [8, 22]. This warrants further work to identify risk factors associated with cardiac decompensation in this population to intervene upon to improve overall outcomes.

## 5 Limitations

The limitations of this study should be noted. As it relies on an administrative dataset, all inherent limitations, including the potential for erroneous coding of diagnosis and procedure codes, are present, with a higher risk due to the complexities of CHD. The absence of detailed clinical data, including liver function tests, cardiac function assessments, echocardiography, pretransplant, and post‐transplant management details, limits the ability to develop comprehensive risk profiles. Additionally, lack of donor data limits our ability to understand donor selection and type of donors. Furthermore, the cohort includes only patients from free‐standing children's hospitals, excluding those from nonchildren's hospitals performing pediatric liver transplants, potentially under‐reporting patients with CHD undergoing liver transplants and may also result in differing graft survival rates compared to other reports. Furthermore, the ability to track patients outside the transplanting facility is limited, potentially leading to an over‐reporting of graft survival.

## 6 Conclusions

Overall, the incidence of a CHD diagnosis in patients with BA undergoing LT is low, around 5%. Long‐term outcomes are not affected by a CHD diagnosis. While patients with CHD experience a higher level of care, no difference in mortality and LOS was evident. Further work is needed to better understand willingness to transplant and the impact of a CHD diagnosis during the transplant process.

## Acknowledgments

The authors have nothing to report.

## Disclosure

The authors have nothing to report.

## Supporting information


**Data S1** Supporting Information

## Data Availability

The data that support the findings of this study are available from the corresponding author upon reasonable request.
