# Supplementary material for: Outcomes of Liver Transplantation in Patients With Congenital Heart Disease and Biliary Atresia. A Multicenter Analysis
Source: Pediatr Transplant. 2025 May 30;29(5):e70110. doi: 10.1111/petr.70110 (PMC12125496; doi:10.1111/petr.70110)
Supplement: Supplementary file 1 — Data S1 Supporting Information [file PETR-29-e70110-s001.docx]

Supplemental Table 1. Congenital Heart Disease Diagnoses

| CHD Diagnoses | N = 99 / % |
| --- | --- |
| Ventricular Septal Defect (VSD) | 24 (24) |
| Atrial Septal Defect + VSD | 14 (14) |
| Pulmonary Artery Stenosis | 12 (12) |
| Congenital Aortic Stenosis | 12 (12) |
| Tetralogy of Fallot | 6 (6) |
| Pulmonary Artery Coarctation | 5 (5) |
| Pulmonary Valve Anomaly | 5 (5) |
| Coarctation of the Aorta | 4 (4) |
| Pulmonary Artery Anomaly | 4 (4) |
| Atrioventricular Septal Defect | 3 (3) |
| Double Outlet Right Ventricle | 3 (3) |
| Supra-valvular Aortic Stenosis | 2 (2) |
| Sub-valvular Aortic Stenosis | 1 (1) |
| Interrupted Aortic Arch | 1 (1) |
| Partial Anomalous  Pulmonary Venous Return | 1 (1) |
| Total Anomalous  Pulmonary Venous Return | 1 (1) |
| Transposition of the Great Arteries | 1 (1) |

Supplemental Table 2. Multivariable Linear Model Length of Stay

| Variable | Percent difference % | 95% CI | P value |
| --- | --- | --- | --- |
| CHDa | 29.21 | 11.04 - 50.34 | **<0.001** |
|  |  |  |  |
| Age | -0.33 | -0.40 - -0.26 | **<0.001** |
| Age in Months |  |  |  |
| Sex |  |  |  |
| Female | 1.48 | -5.07 – 8.49 | 0.660 |
| Race |  |  |  |
| White | Ref | Ref | Ref |
| Hispanic | 7.53 | -1.82 – 17.79 | 0.118 |
| Black | 4.93 | -5.62 – 16.66 | 0.373 |
| Other | 1.30 | -6.56 – 16.28 | 0.751 |
| Insurance |  |  |  |
| Private | Ref | Ref | Ref |
| Government | 15.59 | 7.13 – 24.73 | **<0.001** |
| Other | 4.23 | -6.56 – 16.28 | 0.457 |
| Region |  |  |  |
| Midwest | -2.40 | -11.01 – 7.04 | 0.605 |
| Northeast | -10.41 | -18.39 - -1.66 | **0.028** |
| South | -6.18 | -15.41 – 4.06 | 0.227 |
| West | Ref | Ref | Ref |
| Era |  |  |  |
| 2004-2009 | Ref | Ref | Ref |
| 2010-2015 | -6.46 | -15.82 – 3.93 | 0.214 |
| 2016-2023 | 8.06 | -2.58 – 19.87 | 0.142 |
|  |  |  |  |
| Prolonged Ventilation | 62.58 | 51.46 – 74.51 | **<0.001** |
